# Predictive factors for early mortality after regorafenib or trifluridine/tipiracil initiation in metastatic colorectal cancer

**DOI:** 10.1093/oncolo/oyag142

**Published:** 2026-04-16

**Authors:** Toshiki Masuishi, Shota Fukuoka, Atsuo Takashima, Yosuke Kumekawa, Kentaro Yamazaki, Kenichi Yoshimura, Toshikazu Moriwaki

**Affiliations:** Department of Clinical Oncology, Aichi Cancer Center Hospital, Aichi, 464-8681, Japan; Department of Gastroenterology, Cancer Institute Hospital Gastroenterology Center, Tokyo, 135-8550, Japan; Gastrointestinal Medical Oncology Division, National Cancer Center Hospital, Tokyo, 104-0045, Japan; Department of Gastroenterology, Saitama Cancer Center, Saitama, 362-0806, Japan; Division of Gastrointestinal Oncology, Shizuoka Cancer Center, Shizuoka, 411-8777, Japan; Center for Integrated Medical Research, Hiroshima University Hospital, Hiroshima University, Hiroshima, 734-8551, Japan; Department of Gastroenterology and Hepatology, Kurashiki Central Hospital, Okayama, 710-8602, Japan

**Keywords:** early mortality, regorafenib, trifluridine/tipiracil, metastatic colorectal cancer

## Abstract

**Background:**

Regorafenib and trifluridine/tipiracil (FTD/TPI) are standard later-line treatments for patients with refractory metastatic colorectal cancer. However, these drugs show limited efficacy in some patients, and inappropriate use may increase toxicity and reduce quality of life. This study aimed to identify clinical predictors of early mortality after regorafenib or FTD/TPI initiation.

**Methods:**

We retrospectively analyzed patients enrolled in the multicenter REGOTAS study. Early mortality was defined as death within 12 weeks of treatment initiation. Variables with *P* < .05 in univariate analysis were entered into a multivariable Cox model for overall survival (OS). Patients alive at 15 weeks (a 3-week margin beyond 12 weeks) were censored.

**Results:**

We analyzed 523 patients (regorafenib, *N* = 212; FTD/TPI, *N* = 311). Independent predictors of early mortality were Eastern Cooperative Oncology Group performance status (≥1), low albumin level (<3.5 g/dL), high C-reactive protein level (≥1.0 mg/dL), and short time from first-line chemotherapy initiation (<18 months). The high-risk group (patients with all four factors, *N* = 35) showed higher mortality and shorter OS than the low-risk group (others, *N* = 488): 12-week mortality rate 40% vs 14%, 14-week mortality rate 60% vs 18%, and median OS: 2.8 months vs 7.8 months (hazard ratio: 3.52; *P*-value < .0001).

**Conclusion:**

Our predictive model may help identify patients at high risk of early mortality after regorafenib or trifluridine/tipiracil initiation and support shared decision-making between clinicians and patients regarding further chemotherapy or best supportive care.

## Introduction

Regorafenib and trifluridine/tipiracil (FTD/TPI) are standard later-line treatments for patients with refractory metastatic colorectal cancer (mCRC).^1^^,^^2^ However, these drugs show limited efficacy in some patients,^1^^,^^2^ and treatment in patients unlikely to benefit may only increase toxicity and reduce quality of life. Identifying patients at high risk of early mortality is therefore essential to guide appropriate use of late-line chemotherapy or best supportive care.

This study aimed to identify clinical predictors of early mortality after regorafenib or FTD/TPI initiation.

## Patients and methods

### Patients

We analyzed individual patient data from 24 institutions in the retrospective REGOTAS study supported by the Japanese Society for Cancer of the Colon and Rectum.^3^ The protocol was approved by institutional review boards of all participating institutions. Because this was a retrospective study, written informed consent for treatment was obtained, and the requirement for additional consent for this analysis was waived. Eligibility criteria included prior use of standard treatments (fluoropyrimidine, oxaliplatin, irinotecan, bevacizumab, and anti-EGFR antibody if *RAS* wild-type) except for regorafenib and FTD/TPI and Eastern Cooperative Oncology Group performance status (ECOG PS) of 0-2.

### Outcome measures and statistical analyses

Early mortality was defined as death within 12 weeks of treatment initiation. The 12-week cutoff was chosen because clinical trials of later-line mCRC often require a life expectancy of ≥12 weeks, representing a clinically meaningful threshold for identifying patients unlikely to benefit from treatment. To account for potential variability in follow-up schedules across institutions, a 3-week margin beyond 12 weeks was applied for censoring. Variables with *P* values of <.05 in univariate analysis were entered into the multivariable model. Candidate variables (reported as prognostic factors) included age, sex, ECOG PS, primary tumor location, primary tumor resection, *RAS* (*KRAS* exon2) status, albumin and C-reactive protein (CRP) levels measured immediately before regorafenib or FTD/TPI initiation, number of metastatic sites, and time from first-line chemotherapy initiation. OS was defined as time from treatment initiation to death from any cause with patients alive or lost to follow-up censored at the date of data cutoff. OS was compared between the high-risk group (patients with all four significant factors identified in the multivariable analysis) and the low-risk group (other patients). Subgroup analyses were conducted separately for regorafenib and FTD/TPI.

## Results

### Patient characteristics

Among 550 patients, 523 met the inclusion criteria (regorafenib, *N* = 212; FTD/TPI, *N* = 311), and 27 were excluded due to missing pretreatment serum albumin or C-reactive protein (CRP) data. Among 523 patients, 254 (48.6%) were ≥ 65 years old (median, 64; range, 29-86); 311 (59.4%) were male; 312 (60.0%) had an ECOG PS of 1 or 2; 117 (22.4%) had right-sided tumors (cecum, ascending colon, or transverse colon); 405 (77.4%) had received primary tumor resection; 259 (50.0%) had *RAS* mutant (including *KRAS* exon2 mutant); 236 (45.1%) had serum albumin < 3.5 g/dL; 238 (45.5%) had CRP ≥ 1.0 mg/dL; 53 (10.1%) had CEA ≥ 5 ng/mL; 178 (34.0%) had ≥ 3 metastatic sites; and 140 (26.8%) had time from initiation of first-line chemotherapy < 18 months.

### Predictive factors for early mortality

In multivariable analysis, independent predictors of early mortality include ECOG PS (≥1) (HR: 1.77; 95%CI, 1.16-2.77; *P*-value = .0074), low albumin level (<3.5 g/dL) (HR: 2.38; 95%CI, 1.52-3.82; *P*-value = .0001), high CRP level (≥1.0 mg/dL) (HR: 2.39; 95%CI, 1.52-3.86; *P*-value = .0001), and short time from first-line chemotherapy initiation (<18 months) (HR: 1.93; 95%CI, 1.29-2.84; *P*-value = .001) (Table 1).

**Table 1.** Predictive factors for early mortality in <12 weeks.

Factors		Univariate analysis		Multivariate analysis	
		HR (95% CI)	*P*-value	HR (95% CI)	*P*-value
**Age**	≥65 vs <65 years	1.34 (0.92-1.95)	0.13		
**Sex**	Male vs Female	1.21 (0.83-1.80)	0.33		
**ECOG PS**	1-2 vs 0	2.21 (1.46-3.44)	0.0001	1.77 (1.16-2.77)	.0074
**Primary tumor location**	Right vs Left	1.35 (0.88-2.02)	0.17		
**Primary tumor resection**	No vs Yes	2.09 (1.40-3.06)	0.0004	1.48 (0.98-2.21)	.06
**Histological type**	G3 vs G1-2	1.69 (0.85-3.01)	0.12		
***RAS* (*KRAS* exon2) status**	Mutant vs Wild-type	1.08 (0.74-1.57)	0.70		
**Albumin levels**	<3.5 vs ≥3.5 IU/L	3.92 (2.61-6.06)	<0.0001	2.38 (1.52-3.82)	.0001
**CRP levels**	≥1.0 vs <1.0 IU/L	4.01 (2.66-6.23)	<0.0001	2.39 (1.52-3.86)	.0001
**Number of metastatic sites**	≥3 vs 1-2	1.47 (1.00-2.14)	0.049	1.15 (0.78-1.67)	.48
**Time from initiation of first-line CTx**	<18 vs ≥18 months	2.17 (1.48-2.15)	0.0001	1.93 (1.29-2.84)	.001

Abbreviations: CRP, C-reactive protein; CTx, chemotherapy.

### Overall survival comparing the high-risk group with the low-risk group

**Figure 1.**
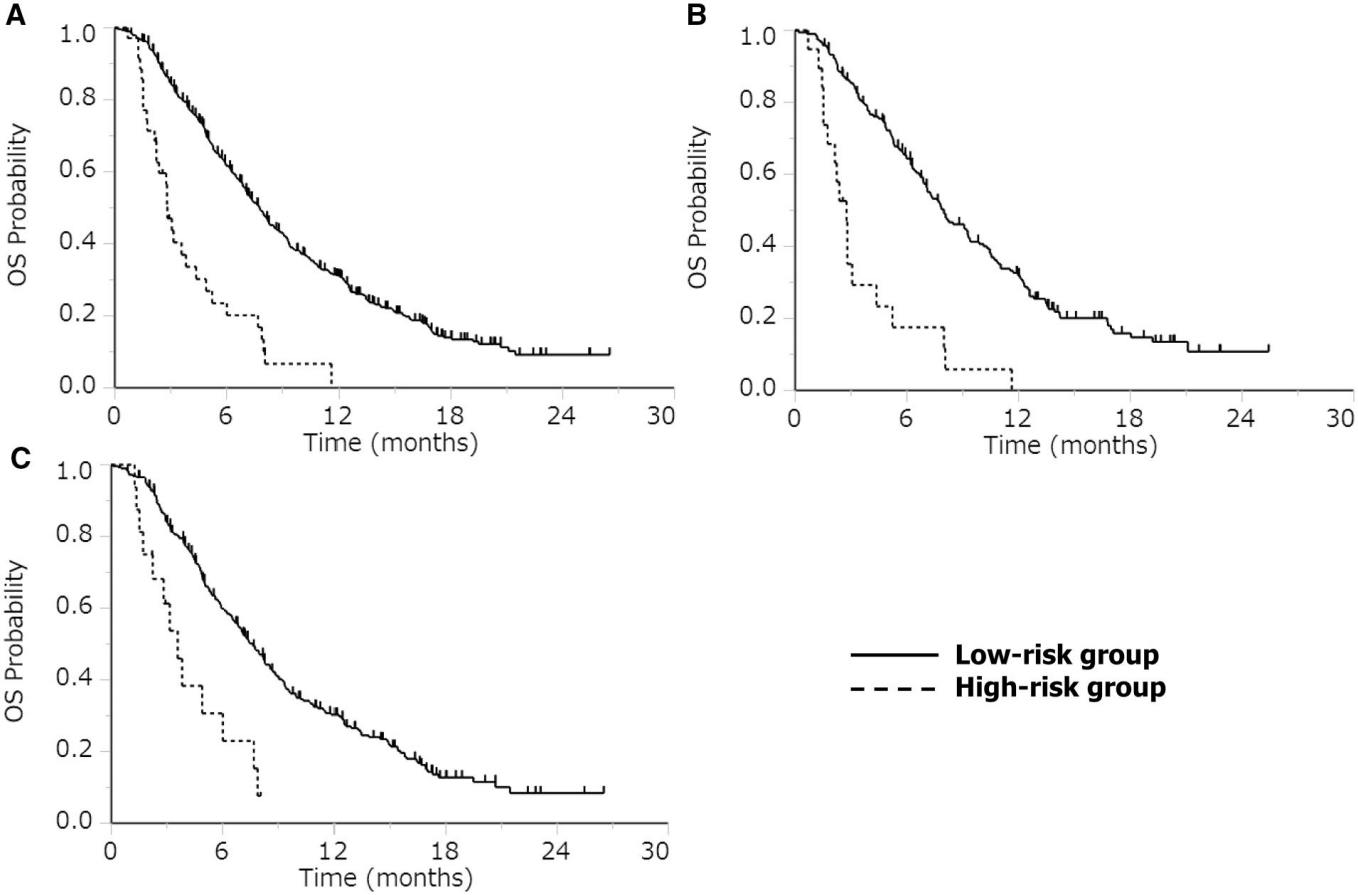
Overall survival for the high-risk and low-risk groups. (A) Median overall survival was 2.8 months and 7.8 months in the high-risk and low-risk groups, respectively. (B) Median overall survival was 2.8 months and 8.0 months in the high-risk and low-risk groups, respectively, in patients treated with regorafenib. (C) Median overall survival was 3.6 months and 7.6 months in the high-risk and low-risk groups, respectively, in patients treated with FTD/TPI.

The high-risk group (*N* = 35) demonstrated higher early mortality rate and shorter OS than the low-risk group (*N* = 488) with 12- week mortality rates of 40% vs 14%, 14-week mortality rates of 60% vs 18%, and median OS of 2.8 vs 7.8 months with HR of 3.52 and *P*-value of <.0001 using the univariate analysis based on Cox proportional hazards model (Figure 1A). The high-risk (*N* = 19) group exhibited higher 12- and 14-week mortality rates and shorter OS than the low-risk group (*N* = 193) for regorafenib (47% vs 14%, 71% vs 16%, and 2.8 vs 8.0 months, respectively) (HR 3.98; *P* < .0001) (Figure 1B). Similarly, the high-risk (*N* = 16) group exhibited higher 12- and 14-week mortality rates and shorter OS than the low-risk group (*N* = 295) for FTD/TPI (32% vs 13%, 46% vs 19%, and 3.6 and 7.6 months, respectively) (HR 3.00; *P* = .001) (Figure 1C).

## Discussion

Determining the optimal transition timing to end-of-life care is challenging for oncologists.^4^ Quality of life (QOL) often deteriorates during later-line treatment because of both tumor- and treatment-related factors.^5^ Identifying patients unlikely to benefit from further chemotherapy is therefore critical for maintaining QOL and dignity near the end of life.

The median OS in the high-risk group was no longer than that in placebo arm of pivotal regorafenib and FTD/TPI trials (5.3 and 5.0 months, respectively).^1^^,^^2^ This finding suggests further chemotherapy may not provide meaningful survival benefit for such high-risk patients.

Prognostic scores such as the NLR^6^ and Royal Marsden score^7^ have been reported, but our model uses simpler and widely available clinical factors. Albumin and CRP are routinely measured in Japan, and our findings may encourage wider use as prognostic markers. Post hoc analyses of RECOURSE suggested that liver metastases and ≥3 metastatic sites were associated with poor outcomes with trifluridine/tipiracil.^8^ As detailed metastatic organ-site data were unavailable, we used the number of metastatic sites as a surrogate for disease burden.

The present study has several limitations. First, this retrospective analysis drew no rigid conclusions. Thus, external validation is warranted. Second, the small number of patients in the high-risk group may limit the statistical power of subgroup analyses. Third, this study included only patients treated with regorafenib or FTD/TPI. Future studies should validate the use of our predictive model for new treatments, including fruquintinib^9^ or FTD/TPI plus bevacizumab.^10^ Fourth, biomarker analysis was not performed.

In conclusion, our predictive model for early mortality after regorafenib or FTD/TPI initiation may be useful for shared decision-making between clinicians and patients, helping decide on further chemotherapy or best supportive care in routine clinical practice.

## Data Availability

To protect the privacy and confidentiality of patients in this study, the datasets generated and/or analyzed during the current study are not publicly available but are available from the corresponding author on reasonable request.
